# Effects of Long Term Supplementation of Anabolic Androgen Steroids on Human Skeletal Muscle

**DOI:** 10.1371/journal.pone.0105330

**Published:** 2014-09-10

**Authors:** Ji-Guo Yu, Patrik Bonnerud, Anders Eriksson, Per S. Stål, Yelverton Tegner, Christer Malm

**Affiliations:** 1 Department of Surgical and Perioperative Sciences, Sports Medicine Unit and School of Sport Sciences, Umeå University, Umeå, Sweden; 2 Department of Health Sciences, Luleå University of Technology, Luleå, Sweden; 3 Winternet, Boden, Sweden; 4 Department of Integrative Medical Biology, Section for Anatomy, Umeå University, Umeå, Sweden; West Virginia University School of Medicine, United States of America

## Abstract

The effects of long-term (over several years) anabolic androgen steroids (AAS) administration on human skeletal muscle are still unclear. In this study, seventeen strength training athletes were recruited and individually interviewed regarding self-administration of banned substances. Ten subjects admitted having taken AAS or AAS derivatives for the past 5 to 15 years (Doped) and the dosage and type of banned substances were recorded. The remaining seven subjects testified to having never used any banned substances (Clean). For all subjects, maximal muscle strength and body composition were tested, and biopsies from the vastus lateralis muscle were obtained. Using histochemistry and immunohistochemistry (IHC), muscle biopsies were evaluated for morphology including fiber type composition, fiber size, capillary variables and myonuclei. Compared with the Clean athletes, the Doped athletes had significantly higher lean leg mass, capillary per fibre and myonuclei per fiber. In contrast, the Doped athletes had significantly lower absolute value in maximal squat force and relative values in maximal squat force (relative to lean body mass, to lean leg mass and to muscle fiber area). Using multivariate statistics, an orthogonal projection of latent structure discriminant analysis (OPLS-DA) model was established, in which the maximal squat force relative to muscle mass and the maximal squat force relative to fiber area, together with capillary density and nuclei density were the most important variables for separating Doped from the Clean athletes (regression  =  0.93 and prediction  =  0.92, p<0.0001). In Doped athletes, AAS dose-dependent increases were observed in lean body mass, muscle fiber area, capillary density and myonuclei density. In conclusion, long term AAS supplementation led to increases in lean leg mass, muscle fiber size and a parallel improvement in muscle strength, and all were dose-dependent. Administration of AAS may induce sustained morphological changes in human skeletal muscle, leading to physical performance enhancement.

## Introduction

Testosterone and other anabolic androgen steroids (AAS) are used by increasing population of professional and recreational athletes with the intention to increase muscle size and improve muscle strength [1]–[3]. Even though athletes using AAS claim significant gain in performance, a large number of academic studies investigating the performance-enhancing effects of AAS have described discordant and often contradictory outcomes [4], [5]. Some studies revealed significant gains in strength and muscle mass/girth [6]–[8] whereas others reported no effects of AAS on muscle mass/girth and/or muscle strength [9], [10]. Such conflicting results have been attributed to poor study design including non-blinded condition, no placebo control, small sample size and AAS dose variation. Above all, in most studies, out of ethic consideration, AAS administration was usually no longer than 6 months. Such a short period of AAS administration obviously could not reflect the reality of AAS abuse in athletes and sport enthusiasts. In reality, AAS usage was estimated to sustain for several years or the whole competition period in athletes [11]. Thus, the difference in AAS administration period between AAS abusers and subjects in most academic studies might be one of the major reasons leading to the different conclusions.

Short term AAS administration has been shown to induce muscle strength enhancement. The increased muscle strength has been attributed to increased muscle mass which was associated with muscle fiber hypertrophy of both type I and type II fibers [12], [13]. Effects of long term AAS administration on muscle morphology in relation with muscle strength as well as with body composition are, however, still unclear. In our early studies on strength training subjects with long period AAS self-administration (9±3.3 years), analysis of muscle biopsies revealed significant increases in mean fiber area for both type I and type II fibers, number of myonuclei and proportion of central nuclei in the steroid users compared to the non-steroid users. In addition, in the steroid users, significant increase in frequency of fibers expressing developmental myosin heavy chain (MyHC) isoforms was also observed compared to the non-steroid users [14], [15]. On basis of the results, we concluded that intake of anabolic steroids in combination with strength training induced both fiber hypertrophy and fiber hyperplasia (formation of new muscle fibres), in which the activation of satellite cells is a key process. However, the studies did not reveal whether the changes in muscle morphology were accompanied by improvement in muscle strength as well as body composition.

In anti-doping campaign, blood and urine samples are the major materials to be tested [5]. However, due to the fast metabolic character of most AAS, remnants of AAS or its metabolites are traceable only for a short time in blood or urine after AAS intake, while the effects of AAS on skeletal muscles will remain for a long period, perhaps lifetime [16]. So far, no study has compared muscle morphology and strength between long-term AAS abusing, and clean athletes.

It has been proposed that the effects of AAS on muscle are dose-dependent [2], [5], [8], [17]. Twenty weeks of testosterone administration increases skeletal muscle mass, leg strength and power in a dose-dependent fashion, but did not improve muscle fatigability or physical function [17]. However, the effects of AAS dosage on skeletal muscles have never been studied over a period of several years.

The present study will investigate the effects of long term supplementation of AAS on muscle strength and morphology, and explore the relationships between AAS dosage, muscle strength and morphology in elite athletes. We proposed that strength training athletes using AAS will have a higher enhancement in muscle strength through morphological adaptations compared with strength training athletes without using AAS. In addition, the effects of long term AAS supplementation on skeletal muscles will be dose-dependent. Thus, the muscular responses to long term AAS supplementation can be detected and used to separate Doped from Clean athletes.

## Materials and Methods

### Ethics Statement

All participants were informed about the design of the study and written informed consent was obtained from all participants. The study protocol was approved by the Ethics Committee for northern Sweden at Umeå University. This was not an intervention study and no actions were taken to influence the participants' exercise training regime, diet, AAS administration or other activities. Manuscript data is confidential and protected by the Swedish personal integrity law (Personuppgiftslagen 1998:204) and the permission from the ethical board for northern Sweden (www.epn.se) at Umeå University (EPN Nr 08–145M).

### Subjects

To investigate the long term effects of AAS supplementation on athletes, we recruited 17 strength training elite athletes through personal contact. All subjects were individually interviewed regarding doping substances, physical activity, smoking habits, known illnesses and medication intake. Ten were current users of AAS or AAS derivatives (Doped; age 41.1±8.0 years) and seven reported that they had never used AAS (Clean; age 29.1±6.2 years). Clean subjects had signed a contract with their local clubs and the Swedish Power Lifting Federation, committing them to never use any drugs, under sever monetary punishment. The subjects have been continuously doping-tested with negative results.

The ten Doped subjects were asked to report all banned-substances including doses and intervals taken for the past years. Detailed information of the banned substances and dosage is shown in Table 1. The AAS administration regimen includes both “stacking” (simultaneous use of several types of AAS) and “cycling” (a drug-free period followed by times when doses and types of drugs taken were initiated or increased). Intake usually follows a pyramid schedule with increased intake over time to avoid equation of AAS levels.

**Table 1:** Self-reported intake of banned substances in the Doped group.

Subject	Substances and dosage in recent 5 years	Substances and dosage >5 years ago
D1	Testosterone (1250 mg w^−1^) Dianabol (8 mg d^−1^) Insulin (10–12 IU d^−1^) IGF I (50 µg d^−1^)	Testosterone (1250 mg w^−1^)Dianabol (8 mg day^−1^) Trenbolone (262.5 mg w^−1^)
D2	Testosterone (2000 mg w^−1^) Deca-durabolin (600–800 mg w^−1^) Dianabol (50 mg d^−1^) Insulin (12 IU d^−1^) Ephedrine (60 mg d^−1^)	Testosterone (2500 mg w^−1^) Insulin (18 IU day^−1^)
D3	Testosterone (1500 mg w^−1^) Deca-durabolin (800–1600 mg w^−1^) Boldone (500 mg w^−1^) Ephedrine (4–6 IU d^−1^, 6 days w^−1^)	Testosterone (1500 mg w^−1^) Deca-durabolin (600 mg w^−1^)
D4	Testosterone 500 mg w^−1^ GH (Somatropin) (4–6 IU d^−1^, 6 days w^−1^) Deca-durabolin (600 mg w^−1^)	Testosterone (500 mg w^−1^) Deca-durabolin (600 mg w^−1^)
D5	Testosterone (500 mg w^−1^) Deca-durabolin (250 mg w^−1^) Dianabol (175–350 mg w^−1^) Ephedrine (10000 IU total)	Testosterone (500 mg w^−1^) Deca-durabolin (250 mg w^−1^) Trenbolone (75 mg w^−1^)
D6	Testosterone (500 mg w^−1^) Deca-durabolin (200 mg w^−1^) Dianabol (200 mg w^−1^)	Testosterone (500 mg w^−1^) Deca-durabolin (200 mg w^−1^)
D7	Testosterone (250 mg w^−1^) Dianabol (175 mg w^−1^)	Testosterone (250 mg w^−1^)
D8	Testosterone (250 mg w^−1^) Deca-durabolin (200 mg w^−1^) Dianabol (200 mg w^−1^) Oxar (175 mg w^−1^)	Testosterone (250 mg w^−1^) Deca-durabolin (200 mg w^−1^) Oxar (175 mg w^−1^)
D9	Testosterone (1000 mg w^−1^) Boldone (1000 mg w^−1^) Dianabol (105 mg w^−1^)	Testosterone (1000 mg w^−1^) Boldone (250 mg w^−1^)
D10	Testosterone (500 mg w^−1^) Deca-durabolin (400 mg w^−1^) Trenbolone (150 mg w^−1^) Dianabol (150–200 mg w^−1^)	Testosterone (500 mg w^−1^) Deca-durabolin (400 mg w^−1^)

All the subjects reported that they had trained regularly between 4–6 times per week for at least five years. The physical training was defined as self-reported mean hours of exercise training each week during the past five years, and consisted mainly of high intensity resistance training. The Doped group consists of a mixture of bodybuilders, strongmen competitors and weightlifters whereas the Clean group consists of weightlifters only. The mode of resistance training differs slightly between the two groups; the Doped group used both 1–4 repetitions/set and 8–12 repetitions/set, while the Clean group used mainly 1–4 repetitions/set. We have to emphasize that this is the only ethically feasible approach to study long term effects of AAS abuse on athletes.

### Muscle strength and body composition

Subjects performed static squats at a 105° knee angle in a custom-made Smith squat machine and ground reaction forces were recorded by AMTI force plates (464 × 508 mm, Advanced Mechanical Technology Incorporated, Massachusetts, USA). Forces were recorded in x, y and z directions at 100 Hz using the Qualisys Track Manager (QTM) software (Qualisys AB, Gothenburg, Sweden). Maximal force (one single recording), Mean of the highest 50 recordings and Mean of 0.1 sec highest recordings according to rank (highest to lowest) were used for statistical analysis. Personal records (PR) from competition (without tight suits) or equivalent (not all participants had competed in all disciplines) for Bench press, Squat lift and Deadlift were also used for comparisons.

### Blood samples

Blood sample of 10 ml was collected from all subjects the same time in the morning after overnight fasting by venipuncture from the cubital vein. Because we could not perform regular doping tests on the subjects and the Doped subjects were not on a “cycle”, indirect indicator of blood hormone level was used to prove/disprove the use of AAS.

### Muscle samples

Skeletal muscle biopsies were obtained from the vastus lateralis muscle using standard needle or forceps biopsy technique [18], [19]. The biopsies were taken from the middle part of the muscle, mounted in OCT compound (Tissue Tek, Miles laboratories, Naperville, IL, USA) and then frozen in liquid propane chilled with liquid nitrogen and stored at −80°C until further processing. Due to technical problems, one biopsy from the Clean group was discarded, leaving 6 biopsies in the Clean group for analysis.

### Biopsy processing

Serial muscle cross-sections were cut at −20°C by using a Reichert Jung cryostat (Leica, Nussloch, Germany). Eight μm thick sections were stained with haematoxylin-eosin and a modified Gomori trichrome staining for basic histopathology including detection of degenerative processes and inflammation [20].

Five μm thick transverse sections were processed for IHC with different and previously characterized antibodies. For fiber phenotype type classification, serial sections were stained with monoclonal antibodies (mAbs) against different MyHC isoforms: A4.840 (strong affinity for MyHCI; [20]), A4.74 (strong affinity for MyHCIIa; [20]), N2.261 (strong affinity to MyHCIIa, weak affinity for MyHCI, no affinity for MyHCIIx; [20]), BF-35 (strong affinity for all MyHC isoforms except IIx; [20]). All antibodies were obtained from the Developmental Studies Hybridoma Bank, developed under the auspices of the National Institute of Child Health and maintained by the University of Biological Sciences, Iowa City, Iowa, USA.

For fiber size measurement and capillary visualization, mAb 5H2 against laminin α2 chain in muscle fiber basement membrane (Nova Castra Lab, Newcastle, UK) and mAb 4C7 against laminin α5 in capillary basement membrane (Chemicon, Temecula, Calif., USA) were used [21].

The IHC staining process is the same as described earlier [22]. Visualization of bound primary antibody was performed using indirect unconjugated immunoperoxidase technique and/or indirect immunofluorescence technique with affinity-purified Abs specifically prepared for multiple labelling and conjugated with flurochromes with different emission spectra, fluorescein (FITC), Rhodamine red-X (RRX) and Cyanine 5 (Cy5). Nuclei were identified with 4′, 6-diamidino-2 phenylindole (DAPI) provided in the mounting media (Vectashield, Vector laboratories Inc, Burlin-game CA 94010 USA). All antibodies were diluted in 0.01 M PBS containing 0.1% bovine serum albumin (Dako, Glostrup, Denmark) and used at their optimal dilution. Control sections were treated similarly except that the primary antibodies were exchanged with non-immune serum.

### Morphometric analysis

Randomly chosen areas from each section were scanned using a light microscope (Leica DM6000B, Leica Microsystems CMS GmbH, Wetzlar, Germany) equipped with a high-speed fluorescence digital CCD camera (Leica DFC360 FX) connected to an image analysis system (Leica, QWin plus). For each muscle sample, more than 50 fibres (mean 227) were individually analysed in order to obtain a robust morphometric analysis [23].

Based on the staining pattern for the different MyHC mAbs, the fibers were classified as fibers containing solely MyHCI, MyHCIIa or MyHCIIx, or as hybrid fibers co-expressing two MyHC isoforms: MyHCI+IIa or MyHCIIa+IIx. Detailed description of fiber type classification has been described in our earlier study [24].

Estimation of fiber area and number of capillary has been described in detail in a publication from our laboratory [21]. An average of 855 capillaries (range 298–1616) per muscle sample cross section was counted. Capillary density (CD) was calculated as the total number of capillaries per μm^2^ muscle cross sectional area (capillary · μm^−2^). The number of capillaries around each fiber (CAF) included all capillaries within a distance of 6 µm from each individual muscle fiber, as outlined by staining for laminin α5. The number of capillaries around each fiber relative to fiber cross sectional area (CAFA) was calculated according to the formula: CAF/(fiber cross sectional area) × 10^3^, representing the cell volume supplied by each capillary.

Nuclei in each fiber (NIF) were calculated as all nuclei within each muscle fiber. The number of nuclei in each fiber relative to fiber area (NIFA) was calculated as: NIF/(cross sectional area for each fiber) × 10^3^. This variable measures the nuclear domain in each fiber. Analysis of internal nuclei in each fiber (INIF) was calculated as all the nuclei within each fiber, but without contact to the cell membrane outline by staining for laminin α5.

### Statistical analysis

Normal distribution of data was tested using the Shapiro-Wilk's test and visually inspected through normal quantile plot. Student's unpaired t-test was used to compare measurements between the two groups, but when data was significantly skewed (p<0.05), then the Wilcoxon signed-rank two-sample test with normal approximation was applied. Accordingly, mean and standard deviation (SD) or median and range were used for descriptive statistics. Correlation analysis between AAS dosage and other variables was performed using Pearson correlation and linear regression, and skew data was log-transformed. Orthogonal projections of latent structures discriminant analysis (OPLS-DA) models were used to separate groups (Clean from Doped). One predictive component was calculated (Y), where R2Y display the cumulative percent of the modelled variation in Y, using the X model. Q2Y values display the cumulative percent of the variation in Y that can be predicted by the model according to cross validation (leave one out methods and seven groups), using the X model. The variation modelled of X, using all predictive components and orthogonal components in X, R2X (cum) is a measure of fit, i.e. how well the model fits the X data. JMP 11 (SAS Institute Inc., USA) and SIMCA 13.0 (Umetrics AB, Sweden) were used for all statistical calculations.

## Results

### Maximal muscle strength and anthropometry

Group values of maximal muscle strength and anthropometry were presented in Table 2. The two groups had similar personal records, but compared to the Clean group, the Doped athletes presented significantly higher lean leg mass (P<0.01) and lower maximal squat force in both absolute and relative terms. For subject G, data of both AAS intake and Type IIa fiber area were outside normal distribution (p<0.05), even after log-transformation. Therefore, results were presented separately with or without the data of the subject.

**Table 2:** Anthropometry, muscle strength and morphology in Clean and Doped athletes [mean ± SD or median (min-max)].

Variable	Doped	Clean	p
**Anthropometry**	N = 10	N = 7	
Body weight (kg)	108±17	110±13	0.85
Lean body mass (kg)	89.8±8.2	74.6±6.8	0.06
Lean leg mass (kg)	28.6±2.5	25.5±1.4	0.01
**Performance**	**N = 9**	**N = 7**	
Personal Bench record (kg)#	205 (155–320)	190 (145–230)	0.79
Personal Squat record	254±11	265±35	0.53
Personal Deadlift record (kg)#	257 (150–300)	269 (245–300)	0.86
Maximal Squat force (N)	2416±633	3302±274	0.004
Maximal Squat force/Lean body mass (N kg^−1^)	29.5±4.0	49.8±5.8	0.001
Maximal Squat force/Lean leg mass (N kg^−1^)	88±17	130±14	<0.001
Maximal Squat force/Mean fiber area (N μm^−2^)	0.33±0.09	0.50±0.05	0.001
Maximal Squat force/Type I fiber area (N μm^−2^)	0.38±0.12	0.64±0.06	<0.001
Maximal Squat force/Type IIa fiber Area (N μm^−2^)	0.28±0.08	0.40±0.06	0.009
**Muscle morphology**	**N = 10**	**N = 6**	
Fiber area (μm^2^)#	7744 (4731–16330)	6733 (5668–8567)	0.70
Type I fiber area (μm^2^)#	6511 (3734–15208)	5189 (4408–6139)	0.30
Type IIa fiber area (μm^2^)#	9066 (4820–17446)	8489 (7144–11448)	0.78
Capillary density (n μm^−2^); CD	218±43	182±41	0.12
Capillaries/Fiber (n); CAF	3.93±0.70	3.05±0.42	0.02
Capillaries/Type I fiber (n); CAFI	4.24±0.60	3.16±0.49	0.003
Capillaries/Type IIa fiber (n); CAFIIa	4.08±0.66	2.94±0.37	0.002
Capillaries/Mean fiber area (n μm^−2^); CAFA	0.55±0.12	0.46±0.11	0.20
Capillaries/Type I fiber area (n μm^−2^); CAFAI	0.69±0.16	0.62±0.11	0.33
Capillaries/Type IIa fiber area (n μm^−2^); CAFAIIa	0.45±0.10	0.36±0.09	0.09
Nuclei/Type I fiber (n); NIFI	2.20±0.11	1.83±0.13	0.04
Nuclei/Type IIa fiber (n)#; NIFIIa	3.84 (2.5–6.0)	3.34 (2.6–4.1)	0.25
Nuclei/Type I fiber area (n μm^−2^) × 1000; NIFAI	0.37±0.10	0.36±0.08	0.83
Nuclei/Type IIa fiber area (n μm^−2^) × 1000; NIFAIIa	0.46±0.10	0.40±0.06	0.21
Internal nuclei/Fiber (n)#; INIF	0.07 (0.01–0.25)	0.07 (0.01–0.36)	0.98

# Wilcoxon signed rank test [median (min-max)].

### Muscle morphology

Group values of measurements were presented in Table 2. The vastus lateralis muscle was predominated by fibers expressing slow MyHCI and fast MyHCIIa fibers in both groups, and there was no difference in fiber type proportion in the muscle between the two groups. Hybrid fibers co-expressing MyHCIIa+IIx or MyHCI+IIa isoforms were rare (< 3%) in some athletes from both groups.

**Figure 1:**
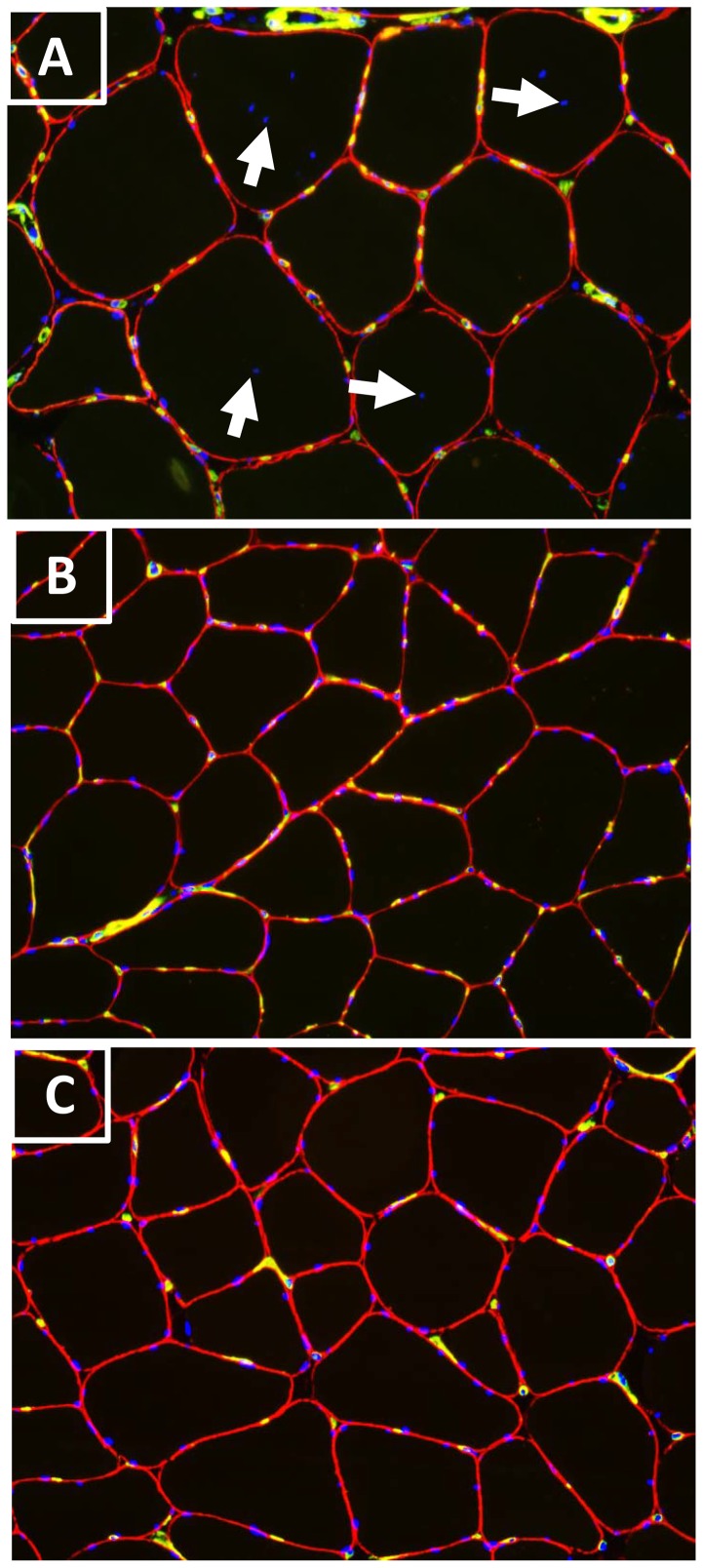
Multiple labeled muscle cross-sections with DAPI (Blue) for nuclei, mAb 4C7 (green) for capillary and mAb 5H2 (red) for fiber membrane. Sections from one Doped athlete using higher (A; >2500 mg·week^−1^) and one using lower doses AAS (B;<500 mg·week^−1^), and from one Clean athlete (C). Doped athletes with higher doses AAS showed larger fiber areas (A) than Doped athletes with lower AAS doses (B) and Clean athletes (C). More capillaries and nuclei around each type I fiber were observed in the Doped athletes (A and B) compared to Clean (C). Internal nuclei are marked with arrows in A.

No significant difference in mean fiber area of either type I or type IIa was observed between the Doped and the lean athletes. However, the doped athletes presented 15% larger in mean fibre area and large variation in fibre area compared to the clean athletes (Fig. 1). In the Doped group, one individual had extremely large fibers (> 15000 µm^2^; Fig. 1).

When capillary measurements were expressed as CD, no difference between the Doped and the Clean groups was observed; however, when expressed as CAF, the Doped group had significantly higher values in both type I and type IIa fibers. When CAF was compensated for fiber area (CAFA), the significant difference between the two groups disappeared for both fiber types (Table 2).

More nuclei per fiber (NIF) were observed in type I fiber of the Doped group compared to the clean group (Table 2). When NIF was compensated for fiber area (NIFA), no difference was observed in any fiber type between the two groups.

The average number of INIF (internal nuclei/fiber) was very low and did not show statistical difference between the two groups.

### Hormone level

Because most blood hormone concentration were not normally distributed, data was analysed by non-parametric statistics (Wilcoxon signed rank, Chi^2^ approximation), and presented as median and minimum - maximum (Table 3). Several blood hormone concentrations linked to hypophysis and liver function differed significantly (p<0.05) between Clean and Doped groups, and were outside clinical ranges. Most notably were LH, where all, and FSH where all but one, Doped subjects had below the clinical range indicating disturbed pituitary gland function. For ASAT and ALAT two, and for CK four, Doped subject were above the clinical range, possibly indicating liver and muscle damage. Seven Doped subjects had testosterone levels above clinical range, but as a group not significantly different from Clean.

**Table 3:** Blood hormone levels [median (min-max)].

*Variable*	*Biomarker for*	Clinical range*	*Control*	*Doped*	*p*
Lutenizing hormone (LH; E • L^−1^)	Pituitary gland	1.2–9.6	2.5 (1.2–4.8)	0 (0–1.2)	<0.001
Folicular stimulating hormone (FSH; E • L^−1^)	Pituitary gland	1.0–12.5	3.0 (1.0–4.2)	0 (0–2.3)	<0.001
17-alfa-Hydroxiprogesteron (17-OH-prog; nmol • L^−1^)	Adrenal glands	< 10	1.85 (0.70–7.00)	0.30 (0.30–0.70)	0.001
Alanine aminotransferas ALAT (μkat • L^−1^)	Liver	< 1.2	0.55 (0.48–0.70)	0.77 (0.59–2.08)	0.005
Aspartate aminotransferas ASAT (μkat • L^−1^)	Liver	< 0.76	0.47 (0.33–0.65)	0.68 (0.39–1.92)	0.01
Prolactin (Prol; μg • L^−1^)	Pituitary gland	3–13	6 (4–13)	10 (6–16)	0.01
Urea (mmol L^−1^)	Muscle/Kidney	3.2–8.1	7.4 (4.5–9.6)	4.8 (2.3–5.7)	0.02
Creatin Kinase (CK; μkat • L^−1^)	Muscle	0.8–6.7	4.8 (3.9–11.8)	11.8 (4.2–85.2)	0.03
Testosterone (Testo; nmol • L^−1^)	Androgen	6.3–16	11 (7.1–18)	35 (3.8–130)	0.08
Pro-brain natriuretic peptide (ProBNP; ng • L^−1^)	Heart	< 84	14 (9–31)	10 (0–32)	0.09
Androstendione (nmol • L^−1^)	Adrenal glands	3.2–9.9	2.0 (1.2–2.8)	3.1 (0.6–14)	0.09
Creatinin (Crea; μmol • L^−1^)	Muscle/Kidney	< 100	94 (84–133)	88 (77–112)	0.12
Apolipoprotein B (ApoB, g • L^−1^)	Liver/Intestine	0.50–1.50	0.87 (0.76–1.44)	1.24 (0.83–1.61)	0.16
Sexual hormone binding globuline (SHBG; nmol • L^−1^)	Liver	15–56	29 (18–46)	23 (3.9–54)	0.19
Apolipoprotein A (ApoA; g • L^−1^)	Liver/Intestine	1.10–1,80	1.19 (1.02–1.60)	1.09 (0–1.36)	0.20
Troponin I (Trop I; μg • L^−1^)	Heart	< 0.03	0 (0–0.11)	0 (0–0)	0.20
Estradiol (E2K; pmol • L^−1^)	Estrogen	50–150	81 (55–102)	182 (25–425)	0.25
Growth hormone (GH; μg • L^−1^)	Hypophysis	none	0.10 (0.01–4.80)	0.05 (0–3.1)	0.33
Insulin like growth factor IGF-I (μg • L^−1^)	Anabolism	120–420	194 (95–384)	158 (114–259)	0.35
Dehydroepiandrosterone sulfate (DHEAS; μmol • L^−1^)	Adrenal glands	2.4–13	8.4 (6.0–11)	7.8 (1.3–12)	0.36
Cystatin C (CystC; mg • L^−1^)	Kidney	< 0.99	0.89 (0.70–0.96)	0.84 (0.75–1.00)	0.59
High sensitive C –reactive protein (HCRP; mg • L^−1^)	Heart/Inflammation	< 3	0.4 (0.3–5–4)	1.37 (0–25.6)	0.73
Lipoprotein (a) (Lp(a); mg • L^−1^)	Heart	< 700	356 (84–1463)	372 (222–782)	0.81
Thyroid stimulating hormone (TSH; mE • L^−1^)	Metabolism	0.4–4.7	1.5 (0.6–2.0)	1.7 (0.8–2.8)	0.85
Cortisol (Cortis; nmol • L^−1^)	Adrenal glands	100–800	415 (281–550)	402 (146–498)	0.87
Alkaline phosphatase (ALP; μkat • L^−1^)	Liver	< 1.20	1.0 (0.7–1.6)	1.15 (0.70–1.70)	0.88

Wilcoxon signed rank test [median (min-max)]. * From the Karolinska University Laboratory (www.karolinska.se/Karolinska-Universitetslaboratoriet/)

### Correlation analysis

**Figure 2:**
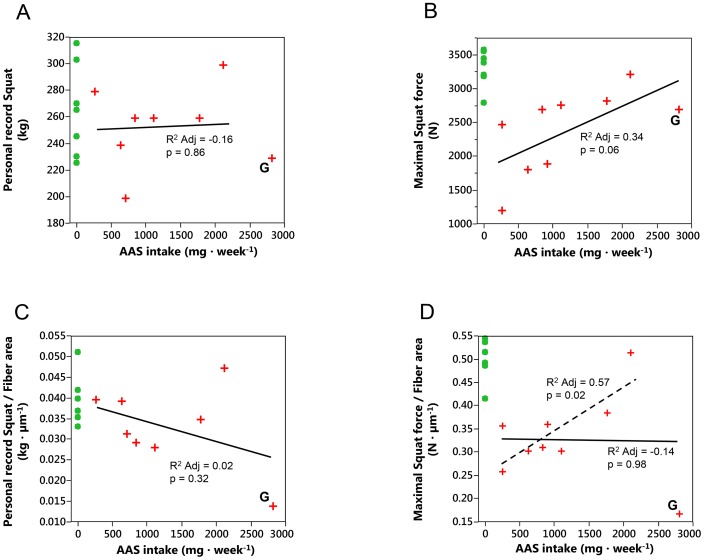
Regression models for the effects of AAS intake on muscle performance. Correlations between AAS weekly intake and muscle performance: A) personal record (kg; R^2^ = −0.16, p = 0.86) and B) maximal squat force (N; R^2^ = 0.34, p = 0.06), and models for the effects of AAS intake on relative muscle performance: C) maximal squat force per lean leg mass (N·g^−1^; R^2^ = 0.02, p = 0.32) and D) maximal squat force per fiber area (N·μm^−1^; R^2^ = −0.14, p = 0.98). The residual of subject G is outlier (p = 0.003, Shapiro-Wilk W test) and when removed, the regression is significant between force per fiber area and AAS intake (N·μm^−1^; R^2^ = 0.57, p = 0.02).

Table 4 was the results of correlation analysis between AAS dosage and all the other measurements. Among the morphological parameters, AAS dose was significantly correlated to fiber area of both type I and type IIa fiber, to CAF of type I, and NIFA of type I fibers. Some linear regression models were presented in Figure 2. A significant regression was found for mean muscle fiber area (R^2^ Adj = 0.40, p = 0.003, not shown), but not for personal record (Fig. 2*A*), nor for personal record relative to fibre area (Fig. 2*C*). A trend (R^2^ Adj = 0.34, p = 0.06) towards significant correlation was observed between AAS intake and maximal squat force (Fig. 2*B*). Subject G had extremely high dose of AAS and skewed regression residuals (p = 0.003). When subject G, the outlier was excluded, a significant linear correlation (R^2^ Adj = 0.57, p = 0.02) between AAS dose and maximal squat force relative to muscle fiber area was observed (Fig. 2*D*). When subject G was excluded, a second-degree fitting reveals a decreasing trend (R^2^ Adj = −0.14, p = 0.98) for maximal squat force relative to fiber area.

**Table 4:** Correlations between AAS dosage and measured variables.

*Variable*	*All subjects (N = 10)*		*Excluding subject G (N = 9)*	
	r	p	r	p
Anthropometry				
Body Weight (kg)	0.33	0.35	0.21	0.59
Lean body mass (kg)	0.62	0.05	0.43	0.25
Lean leg mass (kg)	0.53	0.12	0.27	0.48
Performance				
Maximal Squat force (N)	0.65	0.06	0.75	0.03
Maximal Squat force/Lean body mass (N • g^−1^)	0.51	0.17	0.83	0.01
Maximal Squat force/Lean leg mass (N • g^−1^)	0.55	0.13	0.88	0.004
Maximal Squat force/Mean fiber area (N • μm^−2)^	−0.01	0.98	0.76	0.01
Maximal Squat force/Type I fiber area (N • μm^−2^)	−0.01	0.98	0.80	0.02
Maximal Squat force/Type IIa fiber Area (N • μm^−2^)	−0.08	0.85	0.60	0.12
Morphology				
Mean fiber area (μm^2^)^Log10^	0.69	0.02	0.24	0.53
Type I fiber area (μm^2^)^Log10^	0.70	0.06	0.25	0.51
Type IIa fiber area (μm^2^)^Log10^	0.62	0.06	0.24	0.53
Capillary density (μm^2^); CD	−0.33	0.35	0.52	0.15
Capillaries/Fiber (n); CAF	0.47	0.17	0.59	0.10
Capillaries/Type I fiber (n); CAFI	0.64	0.05	0.61	0.08
Capillaries/Type IIa fiber (n); CAFIIa	0.29	0.41	0.49	0.18
Capillaries/Mean fiber area (n • μm^−2^); CAFA	−0.39	0.27	0.57	0.11
Capillaries/Type I fiber area (n • μm^−2^); CAFAI	−0.43	0.22	0.47	0.20
Capillaries/Type IIa fiber area (n • μm^−2^); CAFAIIa	−0.45	0.2	0.45	0.23
Nuclei/Fiber (n); NIF	0.30	0.40	0.21	0.59
Nuclei/Type I fiber (n); NIFI	0.26	0.47	0.14	0.73
Nuclei/Type IIa fiber (n)^LOG^; NIFIIa	0.33	0.36	0.28	0.46
Nuclei/Fiber area (n • μm^−2^); NIFA	−0.63	0.05	−0.11	0.78
Nuclei/Type I fiber area (n μm^−2^); NIFAI	−0.64	0.05	−0.16	0.69
Nuclei/Type IIa fiber area (n • μm^−2^); NIFAIIa	−0.61	0.06	−0.09	0.81
Inner Nuclei/Fiber (n); INIF	0.55	0.10	0.12	0.76

Log_10_; transformed for normal distribution before calculations.

### OPLS-DA model

**Figure 3:**
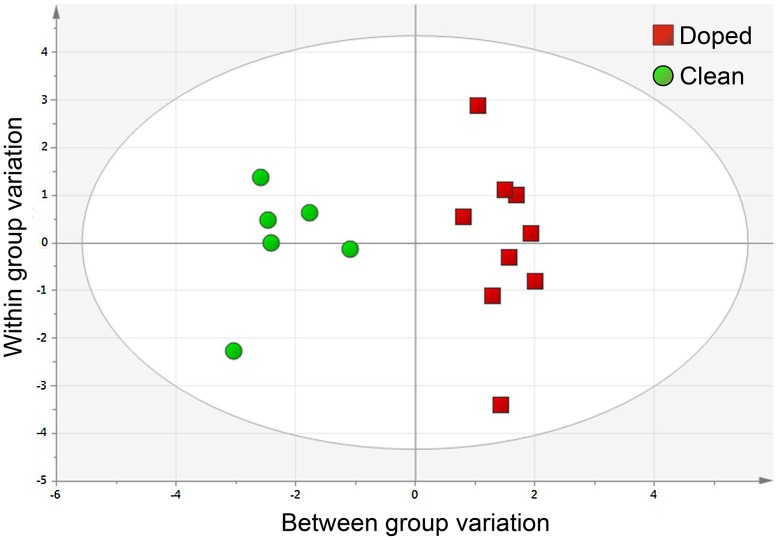
The OPLS-DA model scores scatter plot of combined performance and morphological separates Doped from Clean athletes. Morphological and performance variables (N = 8) are used in an OPLS-DA model to separate Doped (N = 9) from Clean (N = 6) subjects. Regression = 0.93, and prediction by cross-validation = 0.92, p<0.0001, Fisher's exact probability test. All nine Doped subjects and six of seven Clean are correctly classified, leaving one Clean un-classified. Variables of importance are displayed in Figure 4.

**Figure 4:**
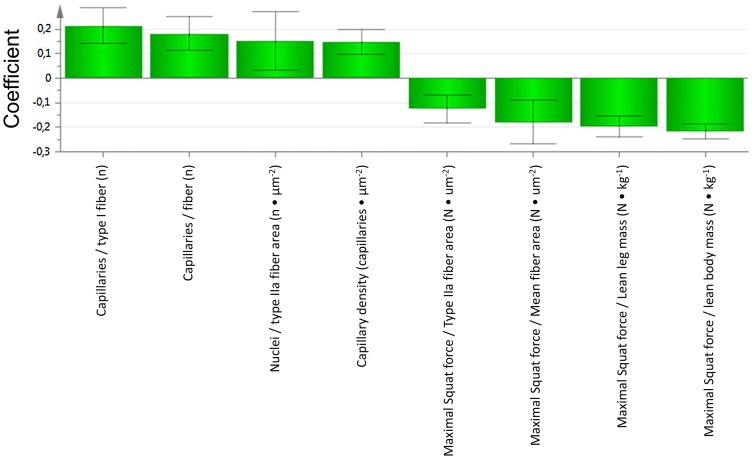
The coefficient plot of variables of importance in the OPLS-DA model. From the OPLS-DA model in Figure 3, the significant important muscle morphological and performance variables separating Doped from Clean subjects are displayed with 95% confidence interval using jack-knifing test. Bars indicate scaled ratios between the Doped and the Clean groups, with higher ratios of the Doped group to the left, and lower ratios to the right.

In OPLS-DA analysis, a score scatter plot (Fig. 3) out of eight morphological and performance measurements separated clearly the Doped from the Clean athletes (Fig. 4). Of the eight variables, four morphological measurements were higher and the other four of relative maximal squat force were lower in the Doped than in the Clean athletes.

## Discussion

The main findings of the study were that the doped athletes had higher lean mass, capillary density and myonuclei density, but lower maximal squat force relative to muscle mass and to fiber area, compared to the clean athletes. The Doped group also had a tendency towards larger fibers, although not significant, most likely due to large variations in fibre area. Low levels of LH and FSH, and high levels of prolactin in some individuals indicate a disturbed pituitary gland function with possible negative effects on reproductive function. High levels of ALAT, ASAT and CK in some individuals suggest that long term use of AAS could damage both liver and muscle tissue. However, no correlations between AAS intake and hormone levels was observed. Thus, testosterone levels at time of sampling cannot explain alternations in these variables, rather concentrations outside clinical limits must stem from long-term supplementation of AAS. Multivariate statistics showed that a combination of eight morphological parameters could clearly separate the doped from the clean athletes. Correlation analysis revealed significant positive correlations between AAS dosage and relative muscle force. The results support previous findings that AAS administration could induce enhancement in both muscle mass and muscle strength, and that the improvements are AAS dose-dependent [5], [8], [12], [25], [26]. This is the first study to confirm previous laboratory findings in active doping athletes.

### AAS and Muscle Mass, Muscle Morphology and Muscle Strength

Despite abundant studies on the effects of AAS on skeletal muscle, many results are contradictory [5]. Some studies have shown gains in body weight, girth, fat-free mass or lean body mass, but not in muscle strength [27]–[29], whereas others have shown gains in both muscle mass/girth and muscle strength [5], [8], [17], [30], [31], or in neither muscle mass/girth nor muscle strength after short term (from 17 days to 16 weeks) [32]–[35] or long term (2 years) [36] AAS administration. The present study showed that athletes with long term AAS supplementation had significantly higher leg lean mass compared to Clean athletes. The higher lean leg mass in the Doped subjects has been revealed to be mainly due to increased muscle mass, as shown in our previous study on the same group of doped athletes [37].

Increased muscle mass in subjects using AAS has been proposed to result from muscle hypertrophy alone [12] or from both muscle hypertrophy and hyperplasia [38]. Muscle hypertrophy is often evident by increased muscle fiber size and increased number of myonuclei. The latter is associated with satellite cell activation and myoblast infusion with the existing muscle fibers, leading to greater numbers of myonuclei in larger myofibers [39]. In previous studies on subjects with long term AAS supplementation (9±3.3 years), we observed significant higher frequency of newly formed myofibers in AAS users than in the non-AAS users, indicating that steroid can induce both muscle hypertrophy and hyperplasia [14], [15]. In the present study, long term AAS supplementation was only associated with higher lean leg mass, but not with larger fiber size, indicating that muscle fiber hyperplasia may play a role in the muscle mass enhancement. Coincidently, the number of myonuclei in type I fibers in the doped athletes was significantly higher than in the clean athletes, which may indicate satellite cell activation for muscle fiber hyperplasia.

Not many studies have examined the effects of AAS on muscle capillaries. In a previous study of 20 weeks of graded testosterone enanthate injection (25, 50, 125, 300, or 600 mg), Sinha-Hikim et al. [12] did not observe significant difference in capillary density among the five treatment groups. The authors suggested that it is not unlikely that a significant increase in capillaries takes longer than 20 weeks. In the present study, we observed more capillaries around both type I and type IIa fibers in the Doped athletes compared to the Clean group. Importantly, when the parameter of capillaries per fiber (CAF) was calculated by fiber area (CAFA), the significant difference in CAF between the two groups disappeared, indicating proportional and simultaneous increases in number of capillary around each fiber and in muscle fiber size in the Doped group. These are the first results demonstrating an association between long term AAS supplementation and muscle capillarization. Consequently, AAS will enhance not only muscle strength, but also muscle endurance.

It has been shown that combined administration of androgens and resistance training is associated with greater gains in lean body mass, muscle size, and maximal voluntary strength than either intervention alone [31]. The greater increase in maximal voluntary strength is often attributed to greater increase in lean body mass and/or muscle size [5]. However, some studies using lower AAS doses and shorter supplementation times have shown no gains in muscle strength [31], regardless if lean body mass and muscle size were increased or not [5]. Similarly, in the present study, the Doped group had higher lean leg mass, but lower leg strength. Thus, for long-term AAS abusers, increase in muscle mass/lean body mass may be not directly associated with muscle strength improvement.

It is worth to notice that compared to the Clean group, the Doped group presented larger variations in many of the measurements like leg lean mass (Doped, 24.6–32.6 kg vs. Clean, 22.8–26.9 kg), leg maximal strength (Doped, 1823–3242 N vs. Clean, 2799–3570 N) and muscle fibre size (Doped, 6055–16330 µm^2^ vs. Clean, 5668–8567 µm^2^). Giorgi et al. [40] have reported that much of the gains in body weight and maximum bench press obtained during, and immediately after, 12 weeks of steroid administration and resistance training was lost during a subsequent 12 week period when androgens were not administered. In the present study, not all Doped athletes were in the same “AAS cycle”, indicating that during the study, some of the Doped subjects were taking AAS whereas the others were in a “clean” period, i.e. AAS effects on muscles were stacking in some subjects but diminishing in the others. This may explain, among other factors, the large variations in some of the measurements, and resulted in the non-significant differences between the two groups [2]. Of course, the large variations in AAS dosage may also explain some of the variations.

### AAS dose-dependent muscular adaptations

Previous studies have shown that testosterone administration was associated with a dose-dependent increase in skeletal muscle mass, leg strength and power [2], [17], [41]. However, similar correlation between AAS dosage and leg lean mass (or fat free body mass) was not observed in the present study. One previous study has shown that 180 days of transdermal testosterone treatment resulted in increase in leg press by 90 days but did not induce further improvement by 180 days [42]. Another study by [43] has shown that major effects of AAS on muscle strength and lean body mass occurred over the first 12 months of testosterone administration to older men. In the present study, because the Doped athletes were not in the same AAS intake “cycle”, the time-dependent effects of AAS on muscles may explain some of the variations in data [2].

In line with laboratory intervention studies [12], [26], we observed that AAS dosage was significantly correlated with fiber area and nuclei number (NIFA; Table 4). Some studies have shown more fiber size enhancement in type I fibers than in type IIa fibers both after short term [44] and long term [14], [45] AAS self-administration. However, our results of fibre size changes in the doped athletes did not show similar fiber type specificity. Finally, if subject G, with extremely high AAS dose, was taken into calculation of correlation between AAS intake and maximal squat force relative to muscle fiber area, there seem to be an upper limit for AAS intake, beyond which further increase in AAS intake will suppress muscular adaptation and performance.

### Study Limitations

While all the Doped athletes have used AAS, the mix and quality of the substance are unknown. This may confound the estimation of AAS dosage as well as the effects on muscle morphology and performance. Additionally, post-study subjects de-coding revealed that Doped group was older and composed of athletes involved in bodybuilding and strongmen events, while Clean athletes were all power-lifters. Consequently, training regiments were slightly different, even though all aiming at increasing muscle strength. Consequences for interpretation of data are several: 1) Doping controls implemented for power-lifters in Sweden has reduced the number of doped athletes, while the same anti-doping efforts have not been taken in other power events. 2) Because the higher age in the Doped group, it can be speculated that athletes in their later career are more prone to AAS. 3) The dose-response effects of AAS on muscle morphology and performance were in agreement with previous intervention studies.

To further explore the effects of long term AAS supplementation on skeletal muscles, more advanced techniques, such as proteomics and metabolomics should be applied in tissue analysing. Again, we have to emphasize that the current study design is hard to be replicated in laboratory due to the extreme doses and duration of AAS supplementation.
